# Is *C. elegans* a suitable model for nutritional science?

**DOI:** 10.1186/s12263-018-0625-3

**Published:** 2019-01-09

**Authors:** Dieter-Christian Gottschling, Frank Döring

**Affiliations:** Department of Human Nutrition and Food Research, University of Kiel, Heinrich-Hecht-Platz 10, 24118 Kiel, Germany

**Keywords:** *C. elegans*, Model, Nutritional biology, Biochemistry of nutrition, Discovery function, Heuristic function, Genetic methods

## Abstract

The suitability of *C. elegans* as a model for the question of nutritional science is a controversial topic. The discussion makes clear that *C. elegans* is its own best model for revealing, via genetic approaches, biological principles of nutritional behavior, and the biochemical function of vitamins. In this case, the model has a discovery function. Worm research serves also in the identification of nutrition-dependent pathways that could be used for novel approaches in human nutritional studies. This heuristic function of the model guides the applied nutrition research in an innovative direction. Since the nutrition and metabolism for the worm and man differ from each other somewhat strongly, results of nutritional studies in *C. elegans* are not directly applicable to human nutrition. In general, the *C. elegans* model is primarily appropriate for explaining the causality of general species’ nutritional phenotypes. Experience tells us that the analysis of drastic nutritional phenotypes in *C. elegans* has the potential to enrich the canon of knowledge of nutritional science.

## Background

The nematode *Caenorhabditis elegans* is widely used as a model organism for studying specific genetic, biochemical, and environmental factors that affect several adaptive behaviors (e.g., foraging), nutrient sensing, lipid storage, and aging. Nevertheless, the suitability of *C. elegans* as a model for the question of nutritional science still remains controversial.

An appropriate model is a simplified version of reality whose essential characteristics match the facts being examined. The fundamental discipline of nutritional science, nutritional biology, has a goal of explaining species general nutrition-dependent phenotypes [1]. As a starting point, nutritional biology analyses general phenomena that are important for the population and/or the organism. The questions of collective and individual foraging strategies, for example, are relevant to this issue.

The classical area of nutritional science is the biochemistry of nutrition. Its objective is to deliver the foundation for nutritional physiology. Since the relevant enzymes are evolutionarily preserved, the results of nutritional biochemistry have a general application across species. In comparison to nutritional biology, the degree of epistemic reduction in biochemistry is significantly larger.

Great similarities with the biochemistry of nutrition are exhibited by molecular nutrition research, the most modern area of nutritional science. Its purpose is to explain the influence of nutrition on gene expression, transcriptomes, proteomes, and metabolomes.

The *C. elegans* model offers a range of genetic methods that are suitable for nutritional science. The available mutants and sources of *C. elegans* RNA mediated interference (RNAi) are appropriate to identify genes that are important for a certain phenotype induced by a nutritional intervention (i.e., longevity via dietary restriction).

RNAi by feeding *Escherichia coli* expressing target-gene double-stranded RNA (dsRNA) permits quick and effective analysis of gene function after post-transcriptional silencing [2].This method is useful to knock-down a gene of interest (i.e., a gene involved in fat metabolism [3]) or to screen all protein-coding genes with respect to a certain phenotype (i.e., lower or higher fat content [4]). The latter approach is possible, because RNAi-libraries, representing almost all protein-coding genes, are available. Thus, the RNAi feeding approach is one of the most employed approaches to analyze gene functions in *C. elegans* research. From a classical genetic point of view, RNAi knock-down leads to a reduction-of-function of a gene and not to a complete loss of gene function. Therefore, the interpretation of RNAi experiments with respect to pleiotropic effects should be considered.

Through this, the function of a gene is, with respect to a reactive answer, directly examined. This epistatic approach is preferred in order to explain a nutritional phenotype on the level of an organism (a population). In comparison, applying omics technologies, only the transcripts, proteins, and metabolites (whose amounts have changed via a nutrition intervention) can be identified. However, their functional meaning remains unclear and should be explained with the application of mutants or RNAi-treated worms. For this, CRISPR/Cas-9 methods are available which allows the easy production of the desired *C. elegans* mutants [5].

### Is *C. elegans* a suitable model for questions of nutritional biology?

An important area of nutritional biology is the increased foraging activity that is observable when there is a waning supply of food. In *C. elegans*, this adaptive behavior was explained by neural networks and central neurotransmitters (dopamine, serotonin) [6, 7]. Other studies demonstrate that *C. elegans* avoids novel foodstuff and prefers familiar food sources [8]. Responsible for this conservative nutritional preference is a serotonin signaling pathway that is functional in the sensory neurons of *C. elegans*. Recently, in worm research, the question why hungry males chose procreation when faced with the choice of food vs. a female, has been addressed [9]. The respective *sex-over-food*s neurons have been shown to be generated in the adult stage via trans-differentiation of glial cells. The phenotypes of the foraging strategies, as well as the neuronal mechanisms, are present in many species. *C. elegans* is therefore a perfect model for decoding the principles of nutritional behavior. More specifically, the *C. elegans* model has a discovery function. In addition, the entire neuronal wiring diagram of *C. elegans*—the connections and circuitry of all the worm’s neurons—is known. It is therefore expected that, with the help of *C. elegans*, further substantial developments in the explanation of nutritional behavior can be targeted.

The geometry of nutrition is another central area of nutritional biology [10]. Its theory assumes that the nutritional selection of living beings is determined by the quantitative relationship of nutrients to each other (geometry), whereby the optimization of Darwinian fitness plays the most important role. The nutritional biology paradigm has been examined in many species (for example in cockroaches, flies, and mice) including primates and is theoretically sound. For nutritional geometric studies, it is crucial to apply synthetic media as sources of food. Since this is only limited possible with *C. elegans*, the nematode is not suitable as a model for nutritional geometry. In contrast, *D. melanogaster* is a proven model for such research [11]. Accordingly, a model for nutritional biology is appropriate if it has a command of phenotypic traits that appear in many species.

### Is *C. elegans* a suitable model for questions of the biochemistry of nutrition?

A central question in the biochemistry of nutrition deals with the function of micronutrients. Therefore, an ideal model for the biochemistry of nutrition would deal with enzymes that are important for the nutritional physiology of other species. The biochemical function of vitamins and trace elements, that define their vital necessity, is the central focus of nutritional science. Several essential functions of micronutrients are documented from worm to man. For the functional explanation of trace elements and ultra-trace elements, *C. elegans* is not suitable, because of its substantial dificulties in using synthetic media. In comparison, the fly model is well applicable for such studies. Recently, it was shown via *D. melanogaster* that bromine fulfilled a vital function for the integrity of connective tissue [12]. Research that unravels the functions of vitamins in *C. elegans* is surprisingly rare. However, a vitamin B_12_-independent pathway for the degradation of uneven numbered fatty acids was identified in *C. elegans* [13]. This alternative metabolic pathway was activated via a regulation of transcription. Such a mechanism might also be important for other species, including humans. *C. elegans* is therefore an appropriate model for understanding the biochemical function and regulation of vitamins. This also applies to other biochemical research areas, for example, the identification of nutrient transporters [14]. In the area of the biochemistry of nutrition, the *C. elegans* model has a discovery function that can be fulfilled mainly through genetic application. For further biochemical analyses of gene products, *C. elegans* is rather inappropriate. Meanwhile, heterologous expression systems are more suitable for this purpose. Nevertheless, for an ideal situation, the genetic applications of *C. elegans* should be combined with those of classical biochemistry.

### Is *C. elegans* a suitable model for questions of molecular nutrition studies?

Molecular nutrition research serves to identify the nutritional relevant signaling pathways. In molecular nutrition research, a model is suitable when a nutritional stimulus (for example, dietary restriction) triggers a signaling pathway that is crucial for many species, including humans. In the past years, there has been a plethora of *C. elegans* studies dealing with the subject of dietary restriction and the effects of secondary plant substances [15, 16]. These studies have discovered important signaling cascades which can be influenced by such nutritional interventions. The signaling pathways identified in *C. elegans* (for example, JNK, AMPK, TOR, autophagy, hormesis) are conserved at molecular level up to humans. Nevertheless, molecular nutrition research is limited when it comes allocating the identified signaling pathway in a physiological situation in humans. Since the physiological differences between worm and human are rather large. Thus, at the level of nutritionally relevant organs (i.e., fatty tissue and the liver) and hormones (i.e., leptin), the worm is far from being a suitable model in this context. The signaling pathways identified in *C. elegans* can serve as approaches for establishing new questions in human nutrition. From this point of view, molecular nutrition research in *C. elegans* has a heuristic function; means, the model can guide the applied nutrition research in an innovative direction.

### Is *C. elegans* a suitable model for questions of human nutrition?

Questions in the area of human nutrition meet the composition and impact of diets on humans. This biomedically oriented research area aims to explore the prevention and therapeutic potential of nutritional habits, food, and nutrients. A model is suitable for such research when it represents prominent aspects of human nutrition and metabolism.

Bacteria serve the worm, in nature as well as in the laboratory, as a source of nutrition. The protein-carbohydrate-fat (energy %) relationship is about 80:10:10. In comparison to humans, the nutrition of *C. elegans* is protein-rich, fat-, and carbohydrate-poor. While humans jettison the amino nitrogen of protein metabolism as urea, worms do so in the form of ammonium [17]. In addition, the worm has a glyoxylate cycle which serves in the de novo synthesis of glucose from even-numbered fatty acids [17]. This metabolic function is not found in humans. Taking account of these differences, the results of nutrition studies with *C. elegans*, for example, with regard to dietary restriction or the modulation of certain macronutrients (i.e., the effect of increased glucose intake [18]), are only limited applicable to human nutrition. *C. elegans* is therefore not suitable to handle questions of human nutrition. The differences between worm and man are based ultimately on various ecological niches and reproductive strategies of both species.

### Are the methods of *C. elegans* suitable for questions of nutritional science?

A classical genetic approach to *C. elegans* is that of forward genetics. With the use of random mutagenesis, a significant amount of mutants can be produced to be examined with respect to a phenotypic characteristic (i.e., large lipid drops [3]). The genes of such mutants can then be identified by SNP-Mapping and whole genome sequencing including several bioinformatics tools for annotation and analysis. This risky, hypothesis-free, and functional approach is the best way to identify genes which could not otherwise be connected to nutrition phenotypes (i.e., collective foraging). Such studies are a desired outcome of nutrition biology research, even though the *C. elegans* model is predestined for it. It is therefore preferred that the forward genetics approach in nutritional science may be the more strongly favored option.

The diet of *C. elegans* consists mostly of bacteria which grow in laboratories on agar plates. The use of axenic, synthetic, and/or liquid media causes drastic phenotypic changes (i.e., lifespan, fertility) in the worm [19]. Because of this, analyses and comparisons, especially in the use of mutants, become more complex. For the research involving *C. elegans*, it is therefore generally important to use bacteria as unique source of food. In consequence, this limits drastically the quantification of food intake, digestion, and energy balance. The possibilitiy of targeted changes in the bacterial food is also rather limited. Because of these methodical limitations, *C. elegans* is not a suitable model for research in which a clear characterization of nutrition physiology is required. One important exception here is the examination of the functions of vitamins. The use of vitamin-deficient bacteria (e.g., vitamin B_12_-deficient bacteria [13]) induces respective deficiency situations in the worm.

Altogether, the possibility for modulation in food in *C. elegans* research is quite limited. Qualitative changes (i.e., food vs. no food) in the food sources are possible. However, this disadvantage can be, upon closer inspection, an advantage; in *C. elegans*, studies should be designed and further developed to focus mainly on extreme nutritional phenotypes. Experience teaches us that only such model investigations have the power to enrich the canon of knowledge of nutritional science [9].

## Conclusion

In conclusion, the *C. elegans* model is primarily appropriate for explaining the causality of general species’ nutritional phenotypes. Experience tells us that the analysis of drastic nutritional phenotypes in *C. elegans* has the potential to enrich the canon of knowledge of nutritional science. In contrast, the use of the *C. elegans* model for other questions in nutritional science (i.e., biochemistry of nutrition, human nutrition) is rather limited, because the metabolism as well as the nutrient intake differs markedly between worm and man.
